# Transcriptomic profile of early zebrafish PGCs by single cell sequencing

**DOI:** 10.1371/journal.pone.0220364

**Published:** 2019-08-14

**Authors:** Xiaoyuan Zhang, Xintian Li, Ronghong Li, Yunbin Zhang, Yiping Li, Shifeng Li

**Affiliations:** 1 State Key Laboratory of Cell Biology, Shanghai Institute of Biochemistry and Cell Biology, Chinese Academy of Sciences, University of Chinese Academy of Sciences, Shanghai, China; 2 Shanghai Key Laboratory of Molecular Andrology, Shanghai Institute of Biochemistry and Cell Biology, Chinese Academy of Sciences, University of Chinese Academy of Sciences, Shanghai, China; 3 CAS Center for Excellence in Molecular Cell Science, Shanghai Institute of Biochemistry and Cell Biology, Chinese Academy of Sciences, University of Chinese Academy of Sciences, Shanghai, China; Academia Sinica, TAIWAN

## Abstract

Single cell RNA-seq is a powerful and sensitive way to capture the genome-wide gene expression. Here, single cell RNA-seq was utilized to study the transcriptomic profile of early zebrafish PGCs (primordial germ cells) at three different developmental stages. The three stages were 6, 11 and 24 hpf (hours post fertilization). For each developmental stage, three zebrafish PGCs from one embryo were collected, and 9 samples in total were used in this experiment. Single cell RNA-seq results showed that 5099–7376 genes were detected among the 9 samples, and the number of expressed genes decreased as development progressed. Based on the gene expression pattern, samples from 6 and 11 hpf clustered closely, while samples from 24 hpf were more dispersed. By WGCNA (weighted gene co-expression network analysis), the two biggest modules that had inverse gene expression patterns were found to be related to PGC formation or migration. Functional enrichment analysis for these two modules showed that PGCs mainly conducted migration and cell division in early development (6/11 hpf) and translation activity became active in late development (24 hpf). Differentially expressed gene analyses showed that more genes were downregulated than upregulated between two adjacent stages, and genes related to PGC formation or migration reported by previous studies decreased significantly from 11 to 24 hpf. Our results provide base knowledge about zebrafish PGC development at the single cell level and can be further studied by other researchers interested in biological development.

## Introduction

As early as 1930s, the zebrafish (*Danio rerio*) has been used for embryological research, and in 1980s, zebrafish was used as a genetically tractable organism [1, 2]. In the 1990s, thousands of zebrafish mutants related to early embryo development were identified [2]. A comparison of the reference genomes of human and zebrafish shows that about 70% of human genes have orthologs in zebrafish [1], so zebrafish has been widely used to study human genetic diseases, such as pediatric disease, cancer, immunological disease, inflammatory bowel disease and so on [2–5]. In addition, thanks to transparent embryos, small size, external fertilization and easily obtainable fertilized eggs, zebrafish becomes an excellent model organism for studying vertebrate development [2, 3, 6, 7].

PGCs are the earliest germline cells which will differentiate into male or female gametes that can give rise to progeny. Preformation and induction are two general ways for the specification of PGCs, and the specification of zebrafish PGCs belongs to preformation [8–10]. For the zebrafish, some maternal RNAs and proteins which are called germ plasm are unequally divided during later cleavage. As a result, cells having germ plasm will develop into PGCs and cells not having germ plasm will develop into somatic cells [8]. In 1997, Yoon et al. and Olsen et al. discovered the first zebrafish PGC marker *vasa* independently [11, 12], and more markers have since been found, such as *nanos3*, *g1m*, *dnd1*, *tdrd7a* and *gra* [13–17]. In 2005, Blaser et al. successfully constructed a transgenic zebrafish whose PGCs could fluoresce green, and this technique made the separation of PGCs from embryos easy and was soon adopted by lots of researchers [18].

Transcriptome analysis is a useful way to detect dynamic changes of gene expression, and it can provide crucial clues to help understand the processes of embryogenesis and development [2, 19]. RNA-seq has become a state-of-the-art tool for transcriptomic research [20]. It has many advantages over previous techniques, such as RNA *in situ* hybridization, quantitative RT-PCR and microarrays. RNA-seq can determine the expression of thousands of genes accurately in just one experiment and can detect new transcripts effectively [21]. Moreover, RNA-seq can be used for rare materials with the newly emerged single-cell RNA-seq technique, which has already been used for the study of human PGCs [22, 23]. For the zebrafish, RNA-seq has been used for studying zebrafish development on the embryo level [6, 7, 24, 25]. However, little is known about the comprehensive transcriptome dynamics in zebrafish PGCs during various early developmental stages.

In this study, single cell sequencing was used to explore the transcriptomes of zebrafish PGCs at three developmental stages: 6 hpf (shield stage), 11 hpf (3-somite stage) and 24 hpf (prim-5 stage). The number of expressed genes decreased as development progressed, and the gene expression pattern was more alike in the 6 and 11 hpf stages (both were in the process of migration) than in the 6/11 and 24 hpf stages. Both gene network and differentially expressed gene analyses showed that PGCs conducted cell division to increase the cell number during migration and translation became active after reaching the destination of the gonad. In addition, genes related to PGC formation or migration, such as *cxcr4b*, *dnd1* and *tdrd7a*, maintained high expression at 6/11 hpf while their expression decreased significantly from 11 to 24 hpf.

## Results and discussion

### Confirmation of zebrafish PGCs

In this study, the embryos of transgenic zebrafish carrying a *kop-egfp-nanos3-3ʹUTR* inserted fragment in the genome were used to isolate PGCs [18, 26]. Based on green fluorescence, single PGC was picked out manually using a capillary tube for downstream experiments (see Methods for details). Three PGCs were picked out from one embryo at each developmental stage (6, 11 and 24 hpf), so 9 PGCs were collected in total from 3 stages (Table 1).

**Table 1:** General information for the high throughput sequencing.

Library	Total paired reads	Paired reads after quality control	Percent of alignment rate to danRer10
6_1	3039238	2959877	94.43%
6_2	3432500	3281638	93.13%
6_3	3956066	3860735	94.27%
11_1	2831994	2756982	93.07%
11_2	4137110	4013189	93.85%
11_3	4337313	4223965	94.61%
24_1	4161471	4037080	93.83%
24_2	4012972	3902837	93.50%
24_3	5212883	5066259	94.12%

**Fig 1:**
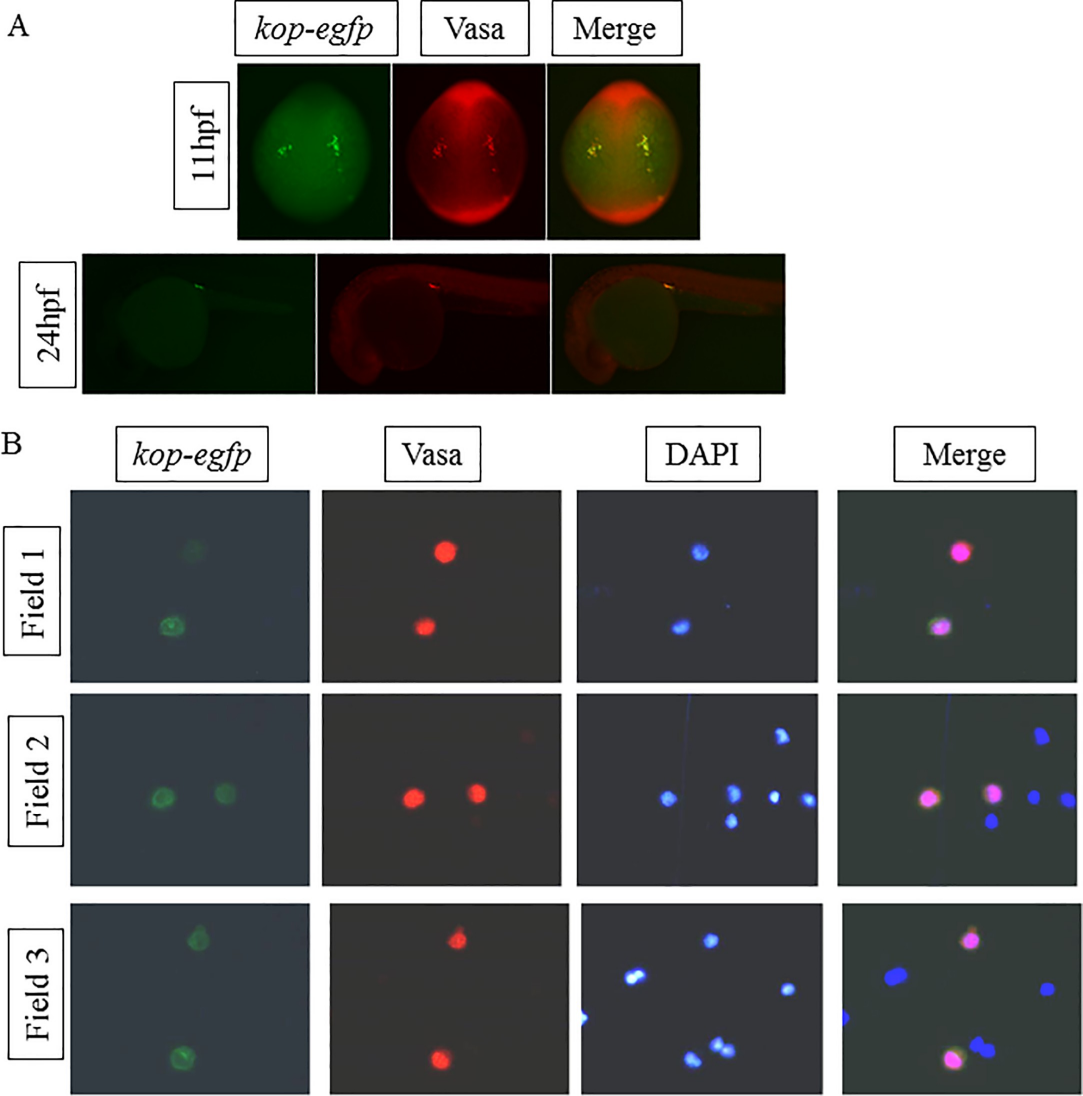
Confirmation of the zebrafish PGCs by immunostaining with Vasa antibody. (A) Whole mount immunostaining with Vasa antibody. Cells containing Vasa proteins can give out red fluorescence (second column). (B) Digested cells from zebrafish gonad at 24 hpf stained by Vasa antibody (second column) and DAPI (third column). Field 1, Field 2 and Field 3 mean three different fields of the glass slide under the microscope.

Under a fluorescent microscope, cells emitting green fluorescence appeared in four locations of the embryo at 6 hpf, formed two relatively loose clusters at 11 hpf and two relatively close clusters in the gonad at 24 hpf (S1 Fig). This result is consistent with previous research about the migration route of zebrafish PGCs [27, 28], and implied that cells emitting green fluorescence might be PGCs. Vasa protein is a typical germline cell marker in zebrafish that has been used to mark PGCs [29–31]. To validate that cells emitting green fluorescence were PGCs, immunostaining with Vasa antibody was used. Images of whole-mount immunofluorescence with Vasa antibody at 11 and 24 hpf showed that cells emitting green fluorescence and cells emitting red fluorescence (stained by Vasa antibody) colocalized perfectly (Fig 1A). Unfortunately, we also did a whole-mount immunofluorescence experiment for the zebrafish embryo at 6 hpf but failed because the red fluorescence was too faint to be detected at this time. In addition, digestive cells from zebrafish gonads at 24 hpf were stained by Vasa antibody and DAPI. Three different fields of a glass slide under the microscope all showed colocalization of cells emitting green fluorescence and red fluorescence (Fig 1B). Above all, we were confident that cells picked out by green fluorescence were PGCs without question.

### Dynamic expression of the PGC transcriptome

**Fig 2:**
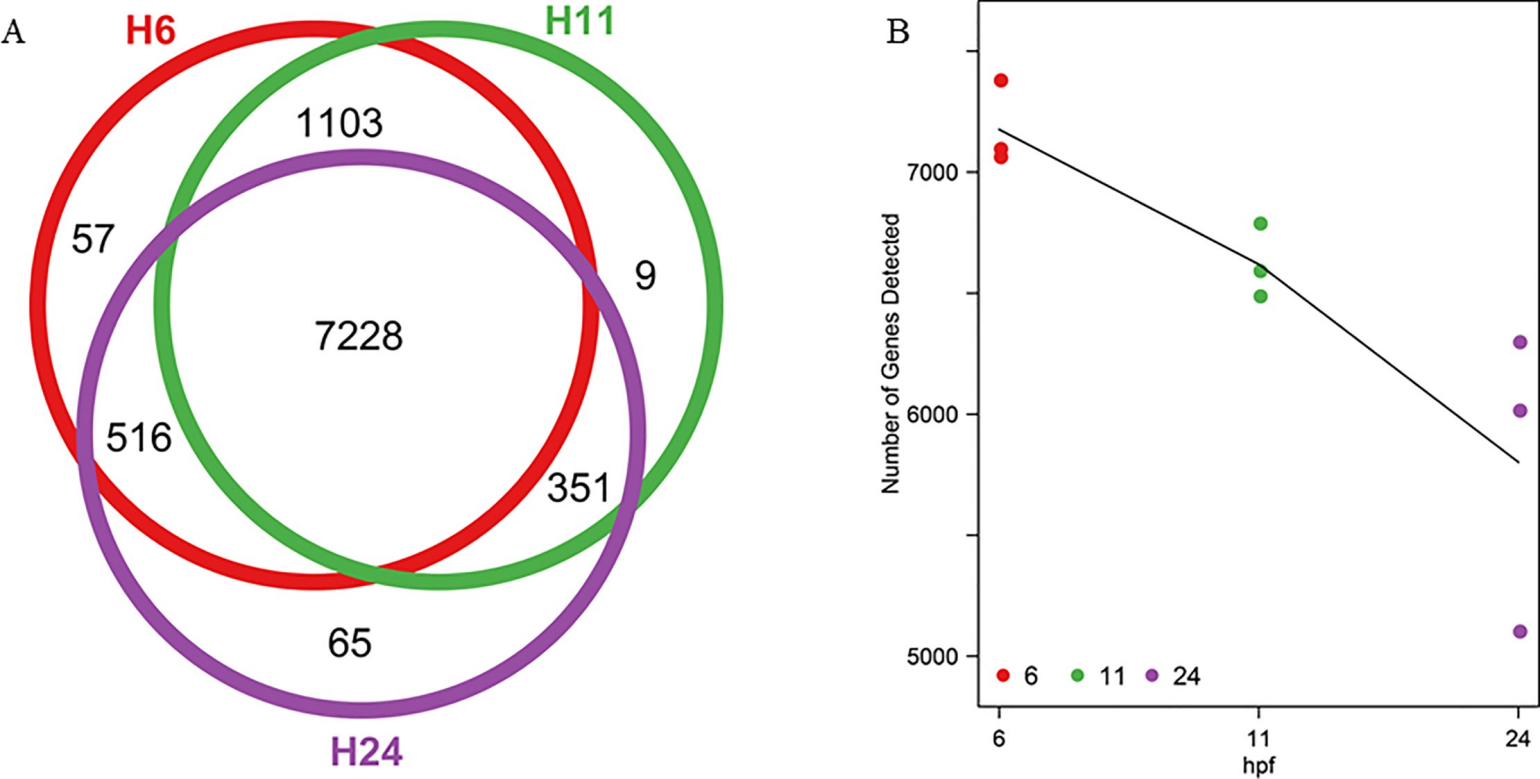
Dynamic changes of the number of genes detected in PGCs. (A) Venn graph for the shared genes at the three developmental stages. (B) Changes of number of genes detected at the three developmental stages.

**Fig 3:**
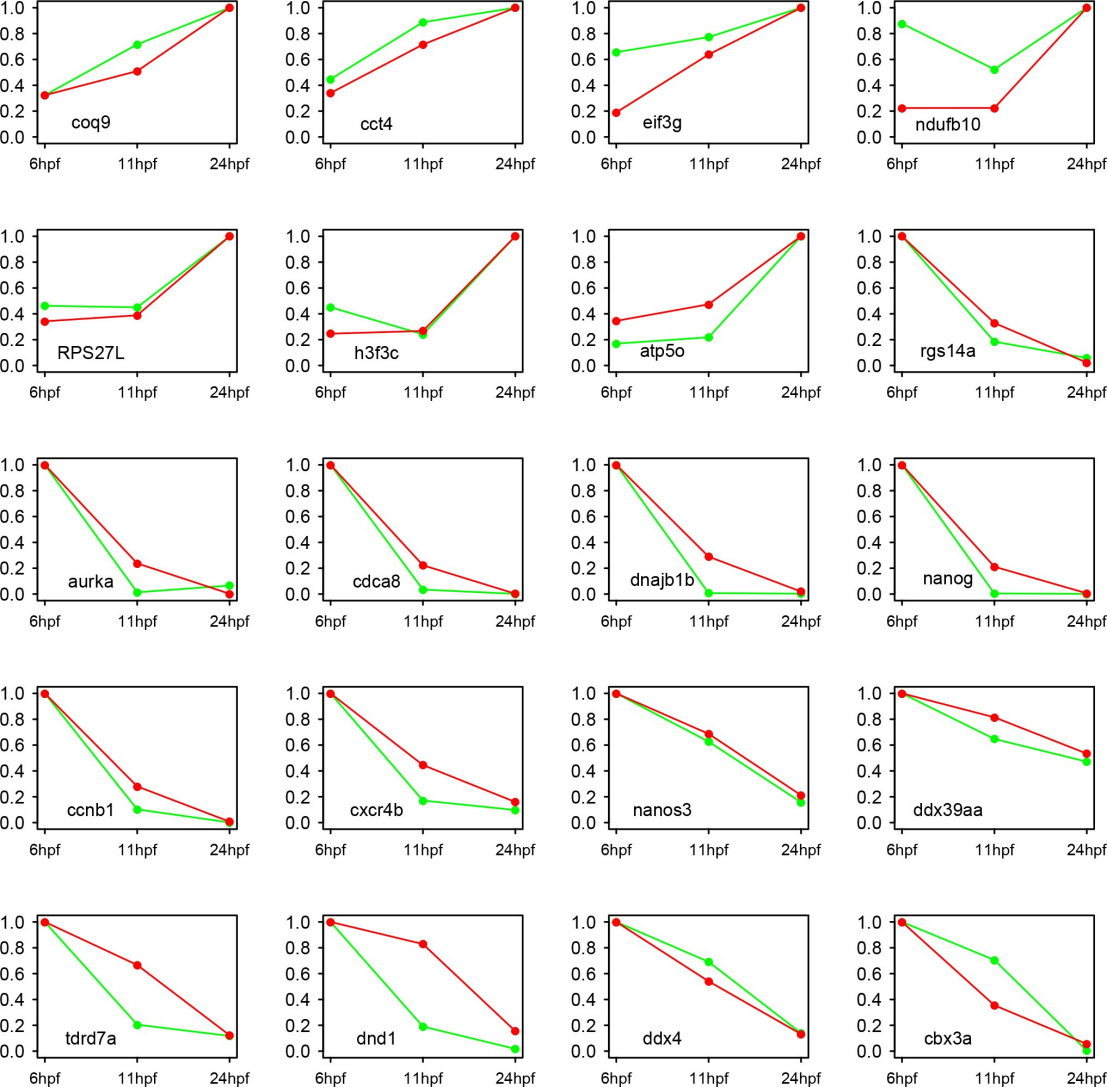
Validation of sequencing data by single-cell qPCR. The relative FPKM values and the relative expression levels of single-cell qPCR are shown. For each gene, the relative FPKM values are calculated as the ratios of the normalized FPKM values to the maximal normalized FPKM value of that gene, where the normalized FPKM values were the mean ratios of the original FPKM values to that of the actb1 gene. The red line indicate the relative FPKM values, the green line indicate the the relative expression levels obtained by single-cell qPCR. actb1 is used as an endogenous control in single-cell qPCR.

To obtain the genome-wide gene expression profile during zebrafish PGC development, nine sequencing libraries were constructed for the Illumina platform with three biological repeats for each development stage. In total, over 35 M paired reads were obtained with about 3–5 M paired reads per individual library (Table 1). A majority of the paired reads were maintained after quality control, and more than 90% of the qualified reads could be mapped to the zebrafish genome (danRer10). The mapped reads were then assigned to specific genes based on the 32,266 annotated genes (danRer10 genome, Ensemble release 89), and gene expression was quantified by calculating the FPKM (Fragments Per Kilobase of exon model per Million mapped fragments) values. To avoid false positives and make the data more effective, genes that had FPKM ≥ 1 at least in three samples were taken into account, and genes not meeting this standard were discarded. Using this standard, 9329 genes remained (about 29% to the total annotated genes) and their expressions were used for the downstream calculation (S1 Table; Fig 2A). Single-cell qPCR was utilized to validate the expression values for 20 selected genes (S2 Fig), and the results were highly consistent with the sequencing data (Fig 3).

The number of genes shared by three developmental stages was 7228, which accounted for more than 77% of the total detected genes, and genes presented only in one stage were less than 70 (Fig 2A). One possible explanation was that these cells were the same kind of cell. Genes detected only in the group of 6 and 11 hpf (1,103) were more than in the two other groups (11 and 24 hpf: 351; 6 and 24 hpf: 516, Fig 2A). Zebrafish PGCs start migration at 4.5 hpf and reach the final destination at 24 hpf [18, 27, 32, 33]. In the stage of 6 and 11 hpf, PGCs are in the processing of migration. At 24 hpf, the migration activity is very weak. So PGCs in the stages of 6 and 11 hpf shared more genes.

With development proceeding, the number of genes detected decreased. PGCs at 11 hpf had 557 fewer genes than at 6 hpf, and PGCs at 24 hpf had 817 fewer genes than at 11 hpf (Fig 2B). This was different from Yang’s result in which the number of detected genes for zebrafish embryos increased steadily during development [6]. Increasingly different kinds of tissues appear during zebrafish embryo development, and the zygotic genome has to transcribe more genes to maintain the function for so many tissues. As a result, genes expressed in the embryo increase with development. In this study, the cells in the three developmental stages are the same kind of cell, and their function is relatively unitary (to form gametes). Maternal transcripts in PGCs degrade with development [34], and gene expression in PGCs is repressed by Dnd1 so as to prevent PGCs from differentiating into somatic cells and maintain germline stem cells [35, 36]. In this study, the least FPKM for Dnd1 was 671, and this value was high enough to suppress the expression of somatic genes. As a result, genes detected in PGCs decreased during development.

### Comparison of transcriptomes at different PGC developmental stages

**Fig 4:**
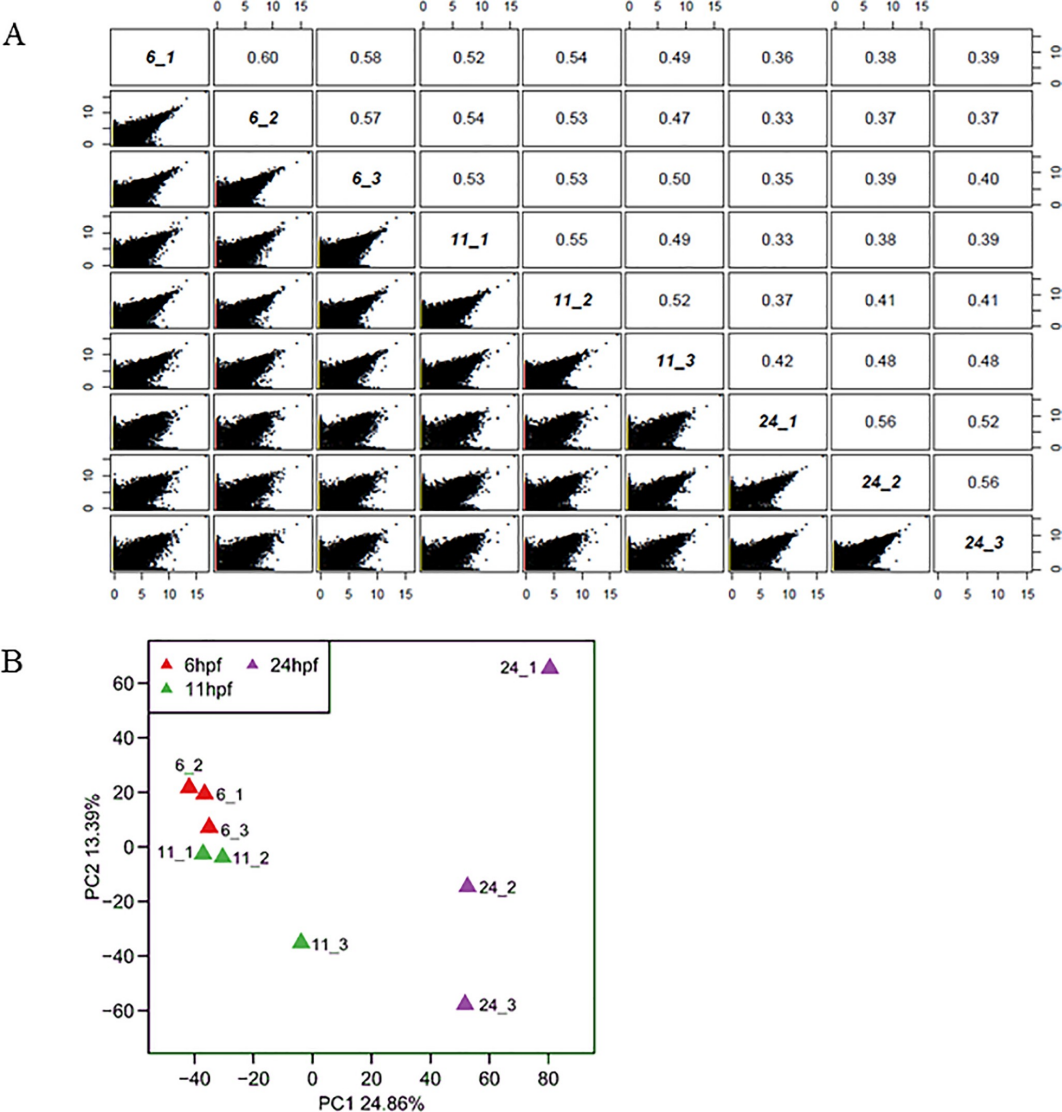
Comparison of gene expression for PGCs at different developmental stages. (A) Pearson correlation analysis between PGCs. The x-axis and y-axis mean gene expression calculated by log_2_(FPKM+1). (B) Principal component analysis for nine PGCs. The first two principal components are displayed on the graph.

To detect similarities in PGCs at different developmental stages, gene expression was compared by Pearson correlation analysis (Fig 4A). The Pearson correlation coefficient (PCC) between any two samples varied from 0.33 to 0.60 with the lowest between 24_1 and 6_2/11_1 and the highest between two samples (6_1 and 6_2) at 6 hpf. PCC between any two samples from the same developmental stage was bigger than 0.50 with one exception between 11_1 and 11_3 (PCC = 0.49). PCC between samples at 6 and 11 hpf was 0.47–0.54, and at 6/11 hpf and 24 hpf was 0.33–0.48. These results showed that individual PGCs were different from each other even at the same developmental stage, and that gene expression pattern of PGCs was more alike at 6 and 11 hpf than at 6/11 and 24 hpf.

Principal component analysis (PCA) was utilized to explore gene expression patterns among PGCs at different developmental stages (Fig 4B). It showed that three samples at 6 hpf and two samples at 11 hpf clustered together closely, while samples at 24 hpf were relatively disperse. This result was further demonstrated by dendrogram analysis (S3 Fig).

In our project, single cell technique was used which required more stringent experimental steps, and minute difference between one cell to another could be captured even though they belonged to the same cell type and were at the same developmental stage. In addition, PGCs at the same developmental stage may really have high variation. For example, Otten et al. found that zebrafish PGCs had highly variable mtDNA copy numbers [37]. As a result, PCC was very low between any two samples even though they were at the same developmental stage (Fig 4A).

Nanog, Pou5f3 and SoxB1 (containing Sox2, Sox3, Sox19a, and Sox19b) are three important transcription factors which can control the expression of thousands of genes in the ealy embryo, and these factors can be used as markers for pluripotent embryonic stem (ES) cell identity [38–43]. In this study, the expression of Sox2 was too low to be detected, and expression of Sox19b/Sox3/Pou5f3/Nanog decreased with the progression of development (S4 Fig). Especially at 24 hpf, the expression of Pou5f3/Nanog was very low, just slightly above zero. The expression of Sox19a reached a maximum at 11 hpf, but the FPKM was just about 20 at this stage. Based on these results, we concluded that at the stage of 6/11 hpf, PGCs had relatively high pluripotency, and all PGCs were in the same developmental states. At the stage of 24 hpf, PGCs had relatively low pluripotency and might have lost synchrony, and some PGCs started to differentiate into downstream germ cells. As a result, dots were more scattered at 24 than at 6/11 hpf in the PCA graph (Fig 4B).

### Gene-network analysis reveals two modules that are important for PGC development

**Fig 5:**
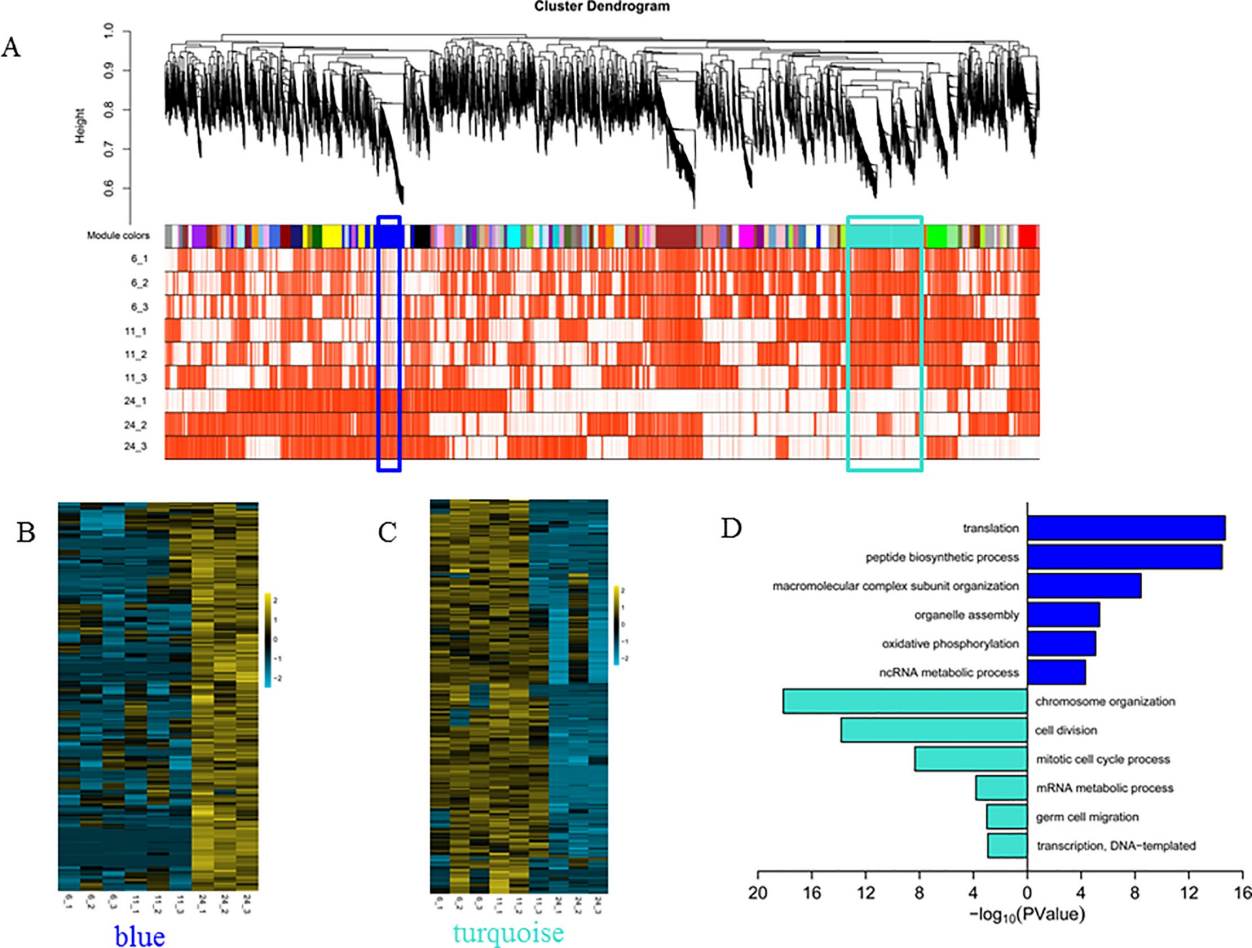
WGCNA for detecting modules related to PGC development. (A) Hierarchical cluster tree for modules identified by WGCNA. The heatmap underneath means gene expression with red for higher and white for lower. The blue module is marked by blue frame and the turquoise module is marked by turquoise frame. (B) Heatmap for gene expresion calculated by log_2_(FPKM+1) and scaled by the row in blue module. (C) Heatmap for gene expresion calculated by log_2_(FPKM+1) and scaled by the row in turquoise module. (D) Functional enrichment analysis for genes in the blue module(blue bar) and the turquoise module(turquoise bar).

Genes having the same expression pattern may contribute to specific biological processes [44]. Here, WGCNA was utilized to investigate gene expression patterns systematically and to find modules of interest [45, 46]. In this study, 103 modules were detected (S2 Table; Fig 5A; a grey module meant that these genes could not be grouped into any specific module). Based on the Pearson correlation between module eigengenes and sample traits (here the trait was hpf), two modules (turquoise and blue) with the biggest absolute PCCs (S5A Fig, turquoise: PCC = -0.91, *p* = 6e-4; blue: PCC = 0.98, *p* = 2e-6) were chosen. These two modules were also the two largest modules (turquoise: 829 genes; blue: 524 genes) of all modules (S2 Table).

For the turquoise module, gene expression was higher in 6/11 hpf than in 24 hpf (Fig 5C). Module eigengene (ME) values for samples in 6/11 hpf were greater than 0.1, except for one sample in 11 hpf (11_3) whose ME value was nearly zero, while ME values for the three repeats in 24 hpf were less than -0.2 (S5C Fig). Some genes that are already known to play an important role in PGC formation or migration belonged to this module, such as *cxcr4b*, *dnd1*, *tdrd7a*, *ca15b* and *ddx4* (S2 Table). Functional enrichment analysis for genes in the turquoise module showed that chromosome organization and cell division process had been enriched, and that germ cell migration and transcription had also been enriched (Fig 5D; S3 Table). These results indicated that genes in the turquoise module were related to PGC proliferation and migration during embryo development.

For the blue module, gene expression pattern was the opposite of that in the turquoise module. Genes in 24 hpf had higher FPKM values than in 6/11 hpf (Fig 5B), and ME values for samples in 24 hpf were greater than 0.4 while those for samples in 6/11 hpf were less than -0.1 (S5B Fig). Functional enrichment analysis for the blue module showed that translation and peptide biosynthetic process were enriched (Fig 5D; S3 Table).

Based on the gene expression pattern and functional enrichment analysis in the turquoise and blue modules, we found that the main task for PGCs were cell division and migration from 6 to 11 hpf, while translation become the main task from 24 hpf and later.

### Differentially expressed genes during PGC development

**Fig 6:**
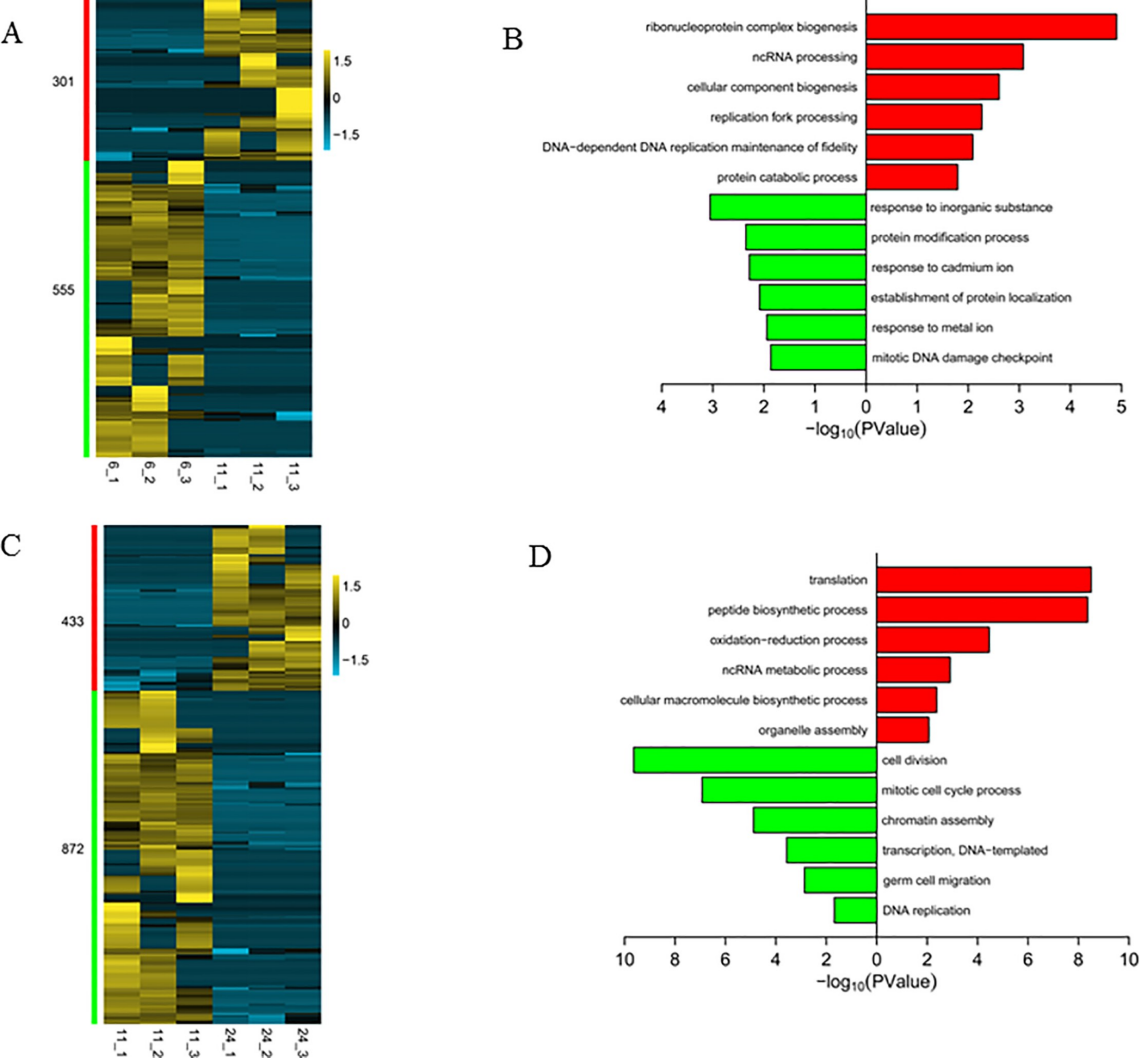
**Expression of differentially expressed genes and their functional enrichment analysis between 6/11 hpf (A,B) and 11/24 hpf (C,D).** Gene expression in the heatmap was calculated by log_2_(FPKM+1) and scaled by the row.

Dynamic changes of differentially expressed genes and the biological processes driven by these genes are useful for studying development. For each adjacent pair of developmental stages, more genes were downregulated. In the pair of 6 and 11 hpf, 301 genes were upregulated and 555 genes were downregulated (Fig 6A; S4 Table). Upregulated genes were enriched in ribonucleoprotein complex biogenesis, cellular component biogenesis and replication fork processing. Downregulated genes were enriched in response to inorganic substance, protein modification process and response to metal ion (Fig 6B; S4 Table). In addition, genes related to PGC formation or migration, such as *cxcr4b*, *dnd1* and *tdrd7a*, were not differentially expressed (S6A Fig), and their expressions remained high during this period (S7 Fig).

In the pair of 11 and 24 hpf, 433 genes were upregulated and 872 genes were downregulated (Fig 6C; S5 Table). Upregulated genes were enriched in translation, peptide biosynthetic process and oxidation-reduction process (Fig 6D; S5 Table), and this corresponded to the blue module in WGCNA (Fig 5D). Downregulated genes were enriched in cell division, transcription and germ cell migration (Fig 6D; S5 Table), and this corresponded to the turquoise module in WGCNA (Fig 5D). Scatter plots and bar graphs showed that genes related to PGC formation or migration, such as *cxcr4b*, *dnd1* and *tdrd7a*, decreased significantly from 11 to 24 hpf (S6B Fig and S7 Fig).

These results again indicated that PGCs conducted cell division to increase their cell number and migration to reach the gonads during early development. After reaching the gonad, the activities of cell division and migration weakened gradually and translation became more active. Knaut et al. showed that the number of PGCs per embryo increases from 15 at 50 percent epiboly to 30 at 24 hpf [30]. Tzung et al. showed that PGC number fluctuates little during the first week of development [47]. Our data are consistent with these previous results, increasing confidence in its validity. Transcription activity weakened from 11 to 24 hpf (Fig 6D), and this was in accordance with the results mentioned above in which the number of genes expressed decreased significantly during this period (Fig 2B). Translation was active from 11 to 24 hpf, and we propose that PGCs were preparing for differentiation during this time because their transcriptomic profiles were more scattered at 24 hpf than at 6/11 hpf (Fig 4B), and gonocytes appeare at 10 dpf (days post fertilization) [48, 49].

## Conclusions

In this study, zebrafish PGCs were separated and sequenced to explore their dynamic transcriptomes during early development. 5099–7376 genes were expressed from 6 to 24 hpf, and the number of genes expressed decreased in the course of development. Gene expression patterns were different between 6/11 hpf and 24 hpf when PGCs were in the process of migration and had finished migration, respectively. Individual differences in PGCs were greater at 24 hpf than at 6/11 hpf, which meant that synchrony was lost at 24 hpf. Functional enrichment analysis showed that PGCs conducted cell division to increase the cell number during migration, and that translation became more active to prepare for differentiation after reaching the final destination of the gonad. Genes related to PGC formation or migration had high expression at 6 and 11 hpf, and their expressions decreased significantly from 11 to 24 hpf. These results help us to understand zebrafish PGC development, and we will focus future studies on zebrafish germ cell development in a wider time range.

## Methods

### Zebrafish strains and breeding

Transgenic AB strain zebrafish carrying the *kop-egfp-nanos3-3ʹUTR* inserted fragment were bought from China Zebrafish Resource Center (CZRC), Wuhan, and bred in the zebrafish platform of Shanghai Institute of Biochemistry and Cell Biology (SIBSB), CAS, under standard breeding conditions [50]. Embryo stages were determined by the description of Kimmel et al [51]. All animal experiments were approved by the Institutional Animal Care and Use Committee of the Shanghai Institute of Biochemistry and Cell Biology, CAS, and all the methods were carried out in accordance with the approved guidelines.

### Isolating single PGC

For zebrafish at 6 and 11 hpf, just one embryo was used respectively. The embryo was dechorionated using forceps, and then single cell suspension was prepared by pipetting the embryo with 20 μL tips for 20 times in a 0.1% BSA solution (dissolved in phosphate-buffered saline, PBS).

For zebrafish at 24hpf, one embryo was collected. The embryo was first anaesthetized by tricaine (3-amino benzoic acid ethyl ester also called ethyl 3-aminobenzoate, Sigma, Cat: A-5040). Then the embryo was dechorionated using forceps, and the embryo head, tail and yolk were cut off with the tip of a 1 mL syringe. The remaining trunk was transferred to a new dish and minced with a syringe tip. Then the minced tissues were pipetted up and down for more than 20 times with 20 μL tips to prepare a single cell suspension.

Single cell suspensions were transferred to slides, and PGCs emitting green fluorescence were picked out using a capillary of 50 μm diameter under an Olympus IX51 microscope. Each isolated PGC was placed in a 200 μL tube with 4.6 μL lysis butter and stored at -80°C. Three PGCs from one embryo were isolated at each stage.

### Whole-mount immunofluorescence

Zebrafish embryos were treated with pronase (Roche, Cat: 10165921001) 1 mg/mL for 10 min at 28°C to lyse the embryo chorion. The dechorionated embryos were fixed with 4% paraformaldehyde/PBS for 1 h at room temperature. Then embryos were fixed with methanol overnight at -20°C and hydrated with gradient methanol (75% 5 min; 50% 5 min; 25% 5 min; PBS 5 min). After that, embryos were washed with PBST (0.1% Triton X-100 in PBS) for 5 min and subsequently blocked with 3% BSA for 30 min. Then the embryos were incubated with anti-Vasa antibody (GeneTex, Cat: GTX128306, 1:1000 dilution) overnight at 4°C. On the next day, embryos were washed with PBST 3 times for 30 min each. Thereafter, embryos were incubated with the secondary antibody (1:500, goat anti-rabbit, Alexa546, Thermo Fisher Scientific, Cat: a11010) for 1 h at room temperature. Finally, the embryos were washed with PBST 3 times for 30 min each and observed by a Zeiss AXIO Zoom V16.

### Immunocytofluorescence

Single cell suspensions of transgenic zebrafish embryo at 24 hpf were prepared as mentioned above. Small circles were drawn on a slide with a Dako pen. One drop of PBS was dropped on the circles and 5 μL of a single cell suspension was transferred into the PBS. After settling for 10 min, cells were fixed with 4% PFA for 30 min at room temperature. Then cells were incubated with 0.5% Triton X-100 in PBS for 15 min, then the cells were blocked with 3% BSA for 30 min and incubated with anti-Vasa antibody (GeneTex, Cat: GTX128306, 1:1000 dilution) overnight at 4°C. Then the cells were washed with PBST 3 times for 5 min each and incubated with the secondary antibody (1:500, goat anti rabbit, Alexa546). Finally, cells were observed by an Olympus IX51 microscope.

### Single-cell qPCR

The single-cell qPCR followed the protocol published before [52]. Briefly, PGC was collected into lysis buffer for the first cDNA synthesis, and a modified Smart2-seq protocol was applied to amplify the cDNA. Then cDNAs which passed quality control were used for qPCR. qPCR was done with NovoStart 480 SYBR qPCR SuperMix Plus (novoprotein) on a LightCycler 480 Instrument (Roche). For the genes at specific developmental stage, five PGCs from one embryo were used and two technical repeats were examined for each PGC. Primers for the 20 selected genes were listed in S6 Table. actb1 is used as an endogenous control.

### RNA-seq library construction and sequencing

The collected PGCs were subjected to Geo-seq [52] for transcriptomic profiling. Briefly, cells were amplified with a modified Smart-seq2 protocol and a library was constructed with Nextera XT kits (Illumina). Sequencing was performed in a 125-bp paired-end format on the Illumina HiSeq 2500. Sequences and processed RNA-seq data files were deposited in the NCBI Gene Expression Omnibus (GEO) database under accession number GEO: GSE122208.

### Read mapping and quantification of gene expression

Raw reads were pre-processed by Trimmomatic version 0.36 [53] and mapped to danRer10 using HISAT2 version 2.0.5 [54]. Gene expression quantification was performed by StringTie version 1.3.3 [54] with the annotation file (Danio_rerio.GRCz10.89.gtf) downloaded from Ensembl. Genes that had FPKM ≥ 1 in at least three samples were taken into account, and genes not meeting this standard were discarded. The Python script prepDE.py (http://ccb.jhu.edu/software/stringtie/index.shtml?t=manual#deseq) was used to calculate the gene read counts for analyzing differentially expressed genes by edgeR.

### Statistical analysis

WGCNA (weighted gene co-expression network analysis) was performed using the WGCNA package [46] in R software [55] with the choice of one-step automatic network construction and module detection. GO (gene ontology) for gene sets of interest was calculated by DAVID 6.8 online [56]. Differentially expressed genes were determined by edgeR [57]. Genes with fold change (FC) ≥ 2 and *p* ≤ 0.05 were treated as differentially expressed genes.

## Supporting information

S1 Fig Images for the zebrafish embryos in this study. White arrows pointing to cells emitting green fluorescence means PGCs. Images of the up panel are taken under bright light, and images of the down panel are taken under fluorescent light.
(TIF)

S2 Fig Results of single-cell qPCR. actb1 is used as an endogenous control, and error bar stands for SE (Standard Error) in 5 PGCs.
(TIF)

S3 Fig Dendrogram analysis for PGCs at different developmental stages. (TIF)

S4 Fig Gene expression of SoxB1, Pou5f3 and Nanog. The y-axis means FPKM values. Sox19a, Sox19b and Sox3 belong to SoxB1
(TIF)

S5 Fig Module eigengene (ME) values for modules identified by WGCNA. (A) Pearson correlation between module engengenes and sample traits (here the trait is hpf). The blue module is marked by blue arrow and the turquoise module is marked by turquoise arrow. (B) ME values for blue module. (C) ME values for turquoise module.
(TIF)

S6 Fig Scatter plots for differentially expressed genes in 6/11 hpf (A) and 11/24 hpf (B). Red dots mean genes upregulated and green dots mean genes downregulated. Genes with fold change (FC) ≥ 2 and p ≤ 0.05 are treated as differentially expressed genes.
(TIF)

S7 Fig Expression of genes related to PGC formation or migration. The y-axis means FPKM values.
(TIF)

S1 Table Gene expression of zebrafish PGCs. These genes have FPKM ≥ 1 in three samples at least, expression values are FPKM values.
(XLSX)

S2 Table Modules detected and gene list in turquoise and blue modules. (XLSX)

S3 Table Functional enrichment analysis by DAVID for gene sets in turquoise and blue modules. (XLSX)

S4 Table Differentially expressed genes in the pair of 6hpf and 11hpf and functional enrichment analysis by DAVID. (XLSX)

S5 Table Differentially expressed genes in the pair of 11 hpf and 24 hpf and functional enrichment analysis by DAVID. (XLSX)

S6 Table Primers for the 20 selected genes. actb1 is used as an endogenous control.
(XLSX)
