# Supplementary figures and images for: Transcriptomic profile of early zebrafish PGCs by single cell sequencing
Source: PLoS One. 2019 Aug 14;14(8):e0220364. doi: 10.1371/journal.pone.0220364 (PMC6693734; doi:10.1371/journal.pone.0220364)

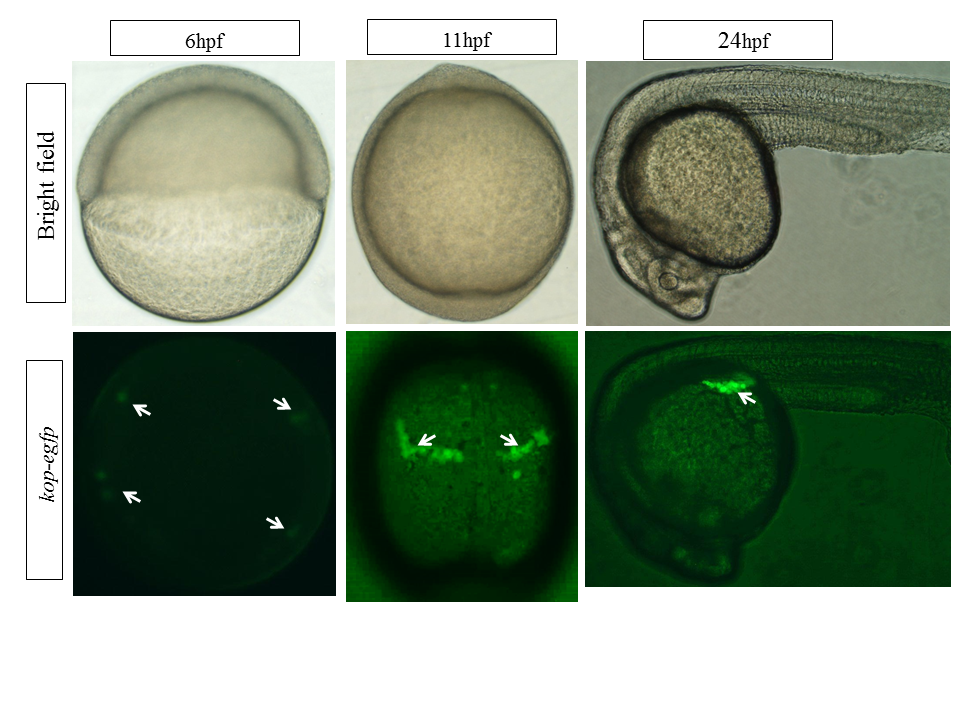

Supplement: S1 Fig — White arrows pointing to cells emitting green fluorescence means PGCs. Images of the up panel are taken under bright light, and images of the down panel are taken under fluorescent light. (TIF) [file pone.0220364.s001.tif]

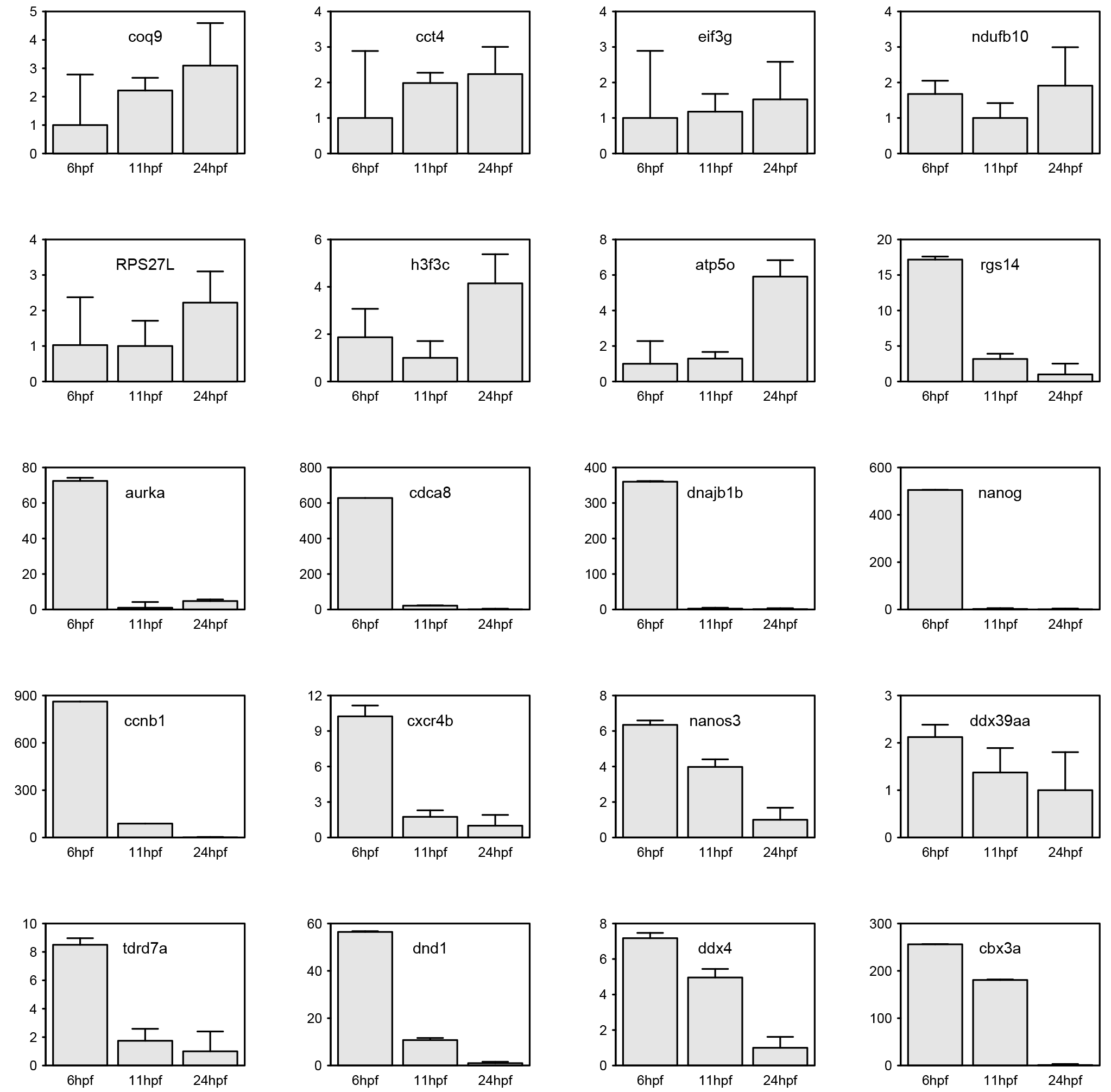

Supplement: S2 Fig — actb1 is used as an endogenous control, and error bar stands for SE (Standard Error) in 5 PGCs. (TIF) [file pone.0220364.s002.tif]

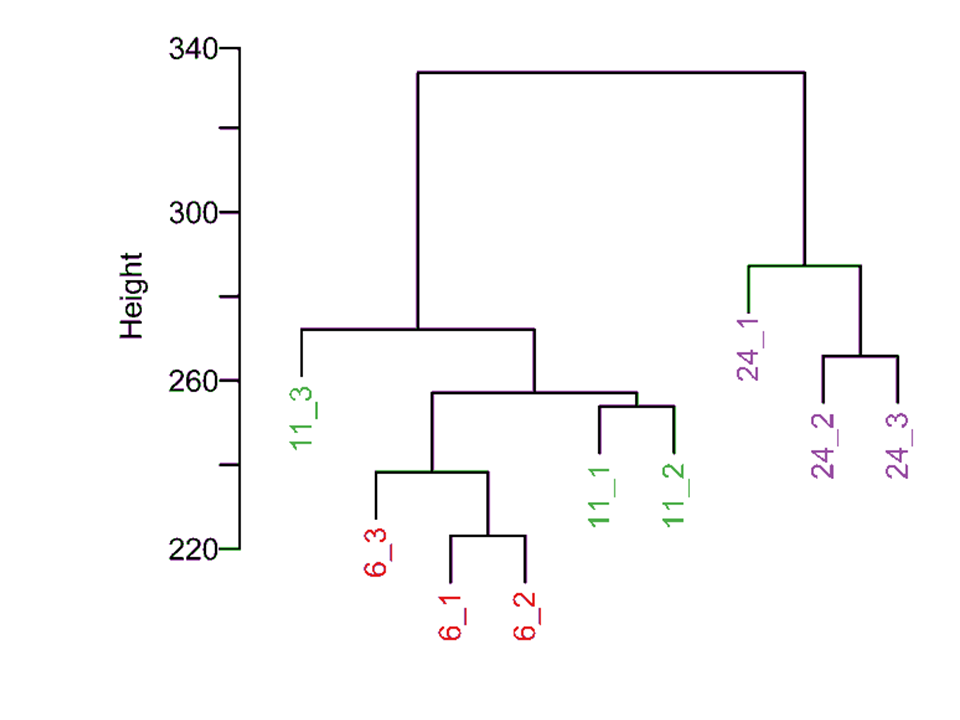

Supplement: S3 Fig — (TIF) [file pone.0220364.s003.tif]

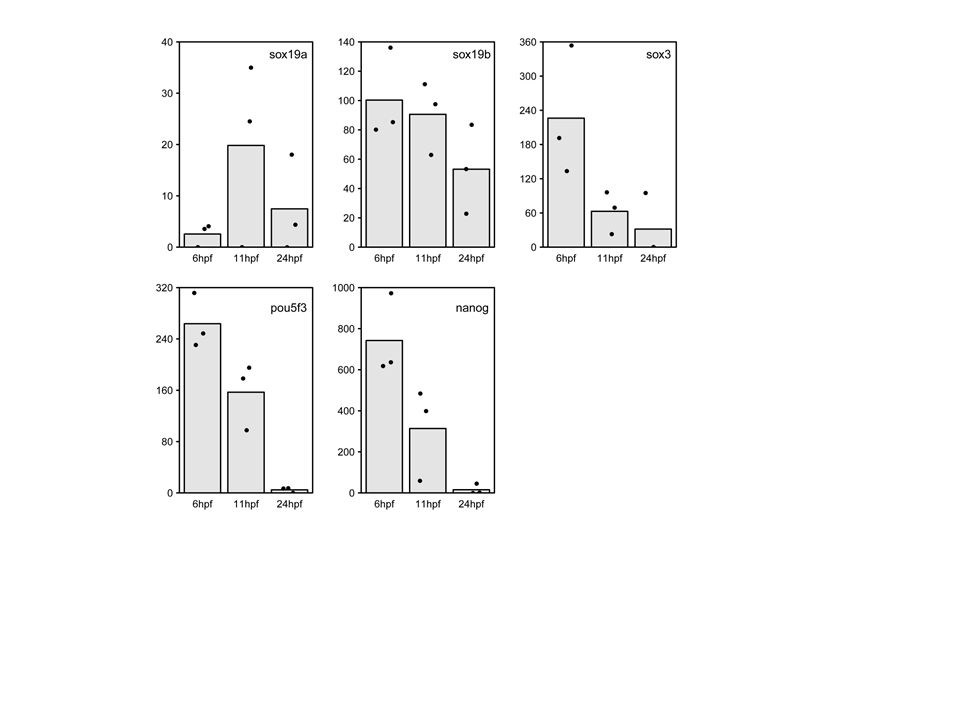

Supplement: S4 Fig — The y-axis means FPKM values. Sox19a, Sox19b and Sox3 belong to SoxB1 (TIF) [file pone.0220364.s004.tif]

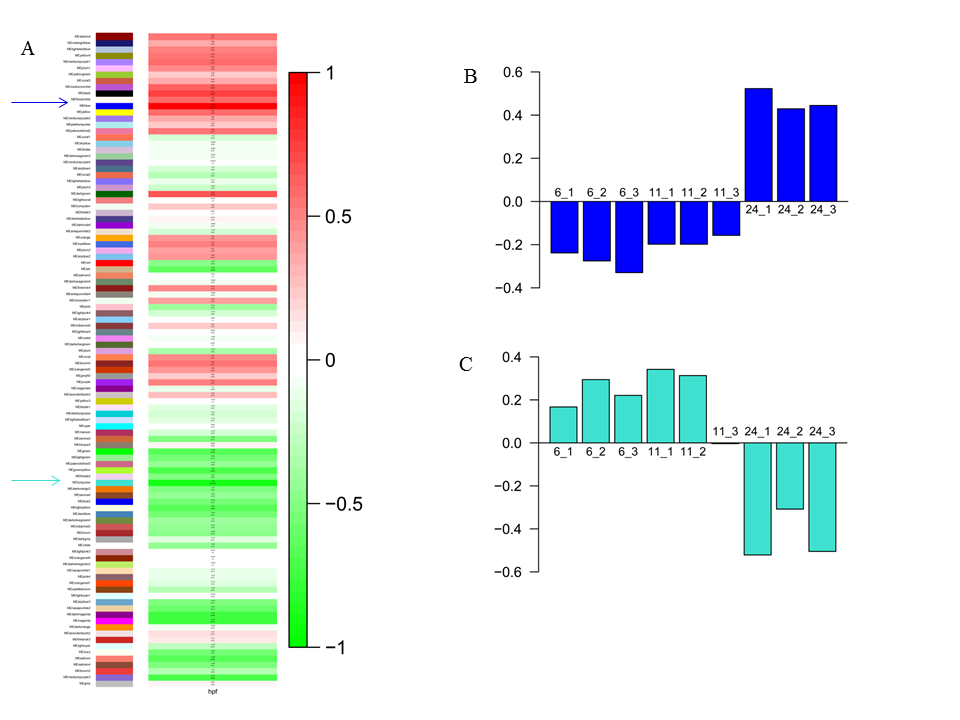

Supplement: S5 Fig — (A) Pearson correlation between module engengenes and sample traits (here the trait is hpf). The blue module is marked by blue arrow and the turquoise module is marked by turquoise arrow. (B) ME values for blue module. (C) ME values for turquoise module. (TIF) [file pone.0220364.s005.tif]

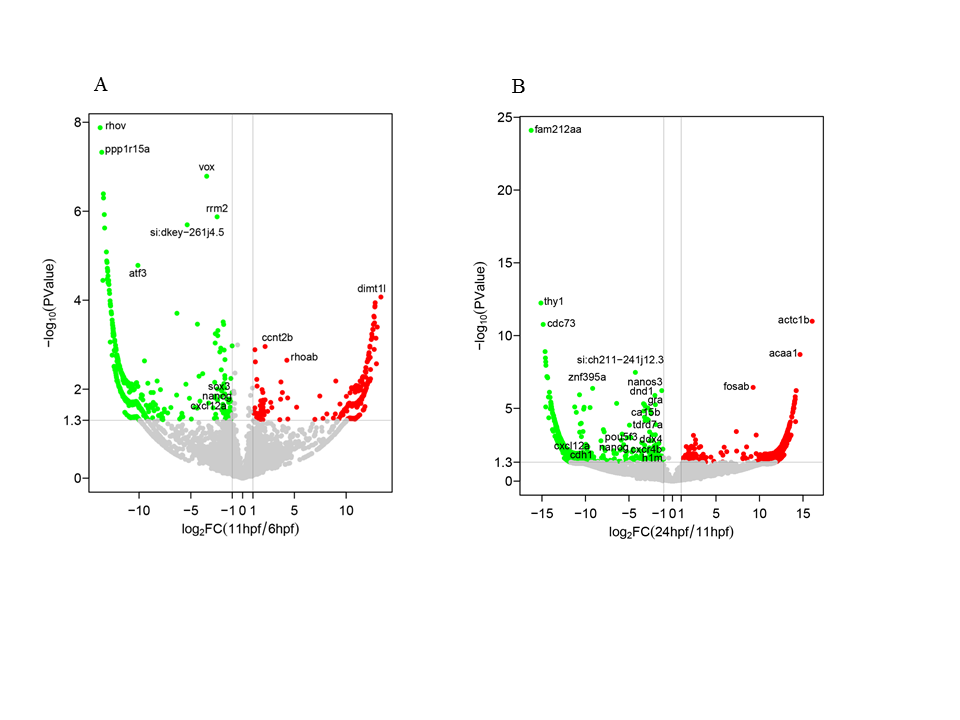

Supplement: S6 Fig — Scatter plots for differentially expressed genes in 6/11 hpf (A) and 11/24 hpf (B). Red dots mean genes upregulated and green dots mean genes downregulated. Genes with fold change (FC) ≥ 2 and p ≤ 0.05 are treated as differentially expressed genes. (TIF) [file pone.0220364.s006.tif]

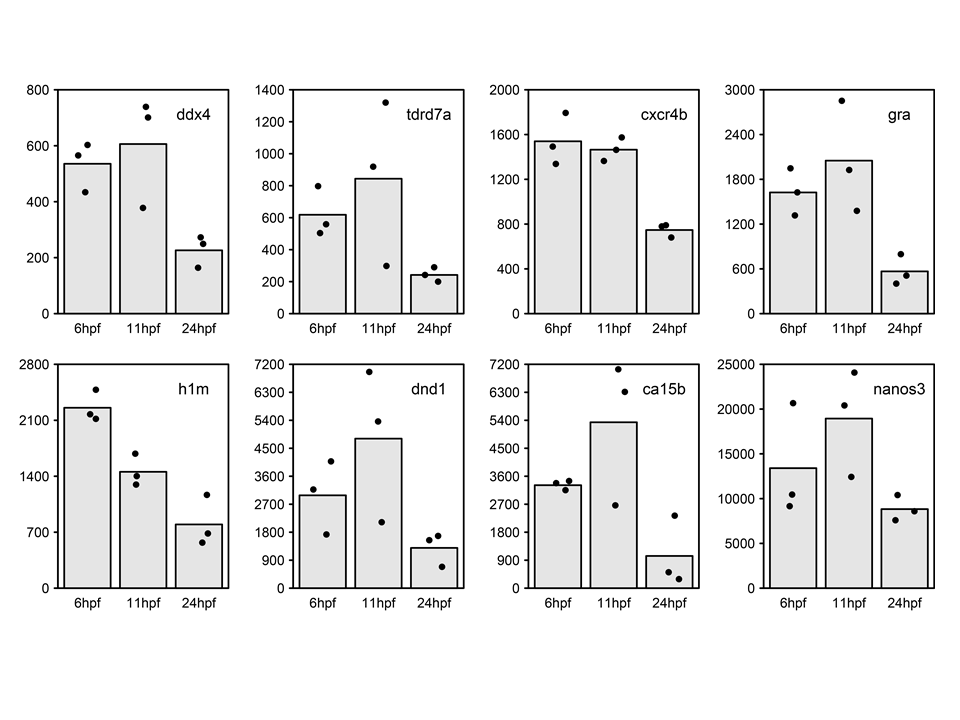

Supplement: S7 Fig — The y-axis means FPKM values. (TIF) [file pone.0220364.s007.tif]
